# Early recurrence in patients undergoing curative resection of colorectal liver oligometastases: identification of its clinical characteristics, risk factors, and prognosis

**DOI:** 10.1007/s00432-017-2538-8

**Published:** 2017-11-11

**Authors:** Junzhong Lin, Jianhong Peng, Yixin Zhao, Baojia Luo, Yujie Zhao, Yuxiang Deng, Qiaoqi Sui, Yuanhong Gao, Zhifan Zeng, Zhenhai Lu, Zhizhong Pan

**Affiliations:** 1 State Key Laboratory of Oncology in South China, Department of Colorectal Surgery, Collaborative Innovation Center for Cancer Medicine, Sun Yat-sen University Cancer Center, 651 Dongfeng Road East, Guangzhou, 510060 People’s Republic of China; 2 Department of Radiation Oncology, Sun Yat-sen University Cancer Center, Guangzhou, 510060 People’s Republic of China

**Keywords:** Colorectal cancer, Oligometastases, Early recurrence, Liver resection, Prognosis

## Abstract

**Purpose:**

Oligometastatic disease can potentially be cured when an optimal approach is performed. Early recurrence after liver resection is an intractable problem, and the clinical implications remain unknown in colorectal liver oligometastases (CLOM) patients. This study aimed to investigate the clinical characteristics, risk factors, and prognosis related to early recurrence in these patients.

**Methods:**

A total of 307 consecutive patients with CLOM undergoing curative liver resection were retrospectively reviewed between September 1999 and June 2016. Early recurrence was defined as any recurrence or death from CLOM that occurred within 6 months of liver resection.

**Results:**

With a median follow-up time of 31.7 months, the 3-year overall survival (OS) and recurrence-free survival rates were 68.7 and 42.5%, respectively. Forty-nine (16.0%) patients developed early recurrence and showed a poorer 3-year OS than those with non-early recurrence (22.3 vs. 75.8%, *P* < 0.001) or later recurrence (22.3 vs. 52.8 vs. 63.2%, *P* < 0.001). Moreover, early recurrence was identified as an independent predictor of 3-year OS [hazard ratio (HR) 6.282; 95% confidence interval (CI) 3.980–9.915, *P* < 0.001]. In multivariate analysis, a node-positive primary tumor [odds ratio (OR) 2.316; 95% CI 1.097–4.892, *P* = 0.028) and metastatic diameter > 3 cm (OR 2.560; 95% CI 1.290–5.078; *P* = 0.007) were shown to be risk factors for early recurrence. The salvage liver resection rate for patients with early recurrence was significantly lower than that for patients with later recurrence (4.1 vs. 19.7%, *P* = 0.010).

**Conclusions:**

Early recurrence should be investigated in routine clinical practice, even in patients with CLOM after curative liver resection. Detailed preoperative comprehensive measurements might help stratify high-risk patients, and a non-surgical treatment for early recurrence might represent an effective alternative.

## Introduction

Colorectal cancer (CRC) has become a leading cause of cancer-related death both in China and worldwide (Chen et al. 2016; Torre et al. 2015). The liver is the most frequent site of metastatic disease. At the time of diagnosis, 20–25% of patients present with synchronous metastases, and approximately half of these patients develop metachronous disease after primary tumor resection (O’Reilly and Poston 2006; Van Cutsem et al. 2010). Despite improvements in the comprehensive treatment and management of patients with colorectal liver metastases (CRLM) in recent years, liver resection remains the most effective treatment, offering the possibility of a cure for CRLM patients (Gallinger et al. 2013; Kanas et al. 2012). Complete liver resection can achieve long-term survival in 46.0% of patients, with a 5-year survival rate of up to 60% (Chan et al. 2014; Kulik et al. 2013). Nevertheless, approximately 60% of patients who first undergo liver resection experience recurrence during follow-up (Chan et al. 2014; Cucchetti et al. 2015). Therefore, identification of different risk subgroups based on the severity of metastatic disease and tumor biological aggressiveness will help establish and optimize therapeutic strategies.

The traditional clinicopathologic factors are inadequate to define the underlying biology of CRLM. In the latest version of the European Society for Medical Oncology (ESMO) Consensus Guidelines, the clinical value of oligometastatic disease (OMD) was highlighted, and metastatic CRC was divided into OMD and widespread systemic disease (Van Cutsem et al. 2016). The concept of OMD emerged 2 decades ago and is typically defined as a state of metastatic disease that is limited in total disease burden, according to the limited number of clinically evident or radiographic sites (Engels et al. 2012; Van den Begin et al. 2014). OMD represents a disease state that exists in a transitional zone between localized and widespread systemic diseases, which shows a genuine potential for cure when patients receive complete R0 resection of their metastases (Reyes and Pienta 2015; Weiser et al. 2013).

It is well known that disease recurrence after liver resection is common and negatively impacts patient survival (Leung et al. 2016; Nordlinger et al. 2008). Early recurrence after liver resection is one of the most important factors for prognosis and quality of life in patients with CRLM. Approximately 10–30% of patients develop early recurrence after liver resection, which is associated with the poorest survival outcome (Imai et al. 2016; Malik et al. 2007; Vigano et al. 2014). To date, few studies have highlighted the clinical implication of early recurrence in colorectal liver oligometastases (CLOM) patients who undergo curative resection. Thus, the aim of this study was to investigate the clinical characteristics, risk factors, and prognoses related to early recurrence after liver resection for patients with CLOM.

## Methods

### Patients and data collection

A total of 413 consecutive patients with CRLM undergoing liver resection between September 1999 and June 2016 at Sun Yat-sen University Cancer Center were retrospectively reviewed. The inclusion criteria were as follows: (1) histologically confirmed adenocarcinoma; (2) colorectal liver oligometastases (≤ 5 metastases); (3) no preoperative extrahepatic metastases; (4) R0 resection for both primary and metastatic tumors; and (5) a minimum follow-up time of 6 months. We excluded 106 patients based on the following exclusion criteria: preoperative extrahepatic metastasis (*n* = 50); R1 or R2 resection (*n* = 37); loss to follow-up (*n* = 2); and number of colorectal liver metastases > 5 (*n* = 17). In total, 307 eligible patients including 176 (57.3%) patients with postoperative recurrence and 131 (42.7%) patients without postoperative recurrence were attentively reviewed for demographic data as well as the tumor characteristics and treatment patterns using an electronic medical record system. The follow-up results were reviewed in detail from the follow-up system at Sun Yat-sen University Cancer Center. All procedures performed in studies involving human participants were in accordance with the 1964 Declaration of Helsinki and its later amendments or comparable ethical standards. Institutional Review Board approval was also obtained from the independent ethics committee at Sun Yat-sen University Cancer Center. Informed consent was waived for this non-interventional, observational, and retrospective study, in which the patient data used were kept strictly confidential.

### Parameter measurements

Primary tumors were staged according to the seventh edition of the UICC-TNM staging system for colorectal cancer. The characteristics of liver metastases, including number, diameter, and distribution, were assessed using enhanced abdominal nuclear magnetic resonance imaging (MRI) at the time of diagnosis. The carcinoembryonic antigen (CEA) and cancer antigen (CA) 199 levels were measured before liver resection. Synchronous metastases were defined as liver metastases diagnosed before colorectal resection or at the time of surgery. The treatment strategy and operability of liver metastases for each patient were determined according to the final agreement of the multidisciplinary team (MDT). Patients considered potentially resectable or at high risk of postoperative recurrence were recommended to receive preoperative chemotherapy first.

After liver resection, patients were monitored through subsequent visits every 3 months for the first 2 years and then semiannually until 5 years. At each clinical review, blood tests were performed for CEA and CA 19-9 levels, along with computed tomography (CT) imaging of the chest, abdomen, and pelvis at 3, 6, 12, and 18 months, 2 years, and annually thereafter. Liver MRI was used to define suspicious lesions indicated on CT or in cases of negative CT results with rising CEA or CA 19-9 levels. The final follow-up visit occurred in June 2017. Overall survival (OS) was defined as the time interval from liver resection to death from any cause or the last follow-up date, while recurrence-free survival (RFS) was defined as the time interval from liver resection to disease recurrence, death from disease, or the last follow-up date. According to the previous data, early recurrence was defined as disease recurrence or death from liver resection within 6 months after liver resection (Jung et al. 2016; Malik et al. 2007). Later recurrence was defined as disease recurrence or death from liver resection at least 6 months after liver resection, including middle recurrence (6–24 months after liver resection) and late recurrence (> 24 months after liver resection).

### Statistical analysis

The statistical analyses were performed using the IBM SPSS Statistics 21 software (IBM, NY, USA) and Graphpad Prism version 6.01 (GraphPad Software, Inc, USA). Values are presented as the median (range) and percentage. The correlation between clinicopathologic parameters and early recurrence was compared using the Chi-square test or Fisher’s exact test as appropriate. Variables that were statistically significant in univariate analysis were further assessed with a logistic regression model for multivariate analysis to identify independent factors associated with early recurrence, and odds ratios (ORs) and 95% confidence intervals (CIs) were subsequently calculated. The OS and RFS rates were estimated with the Kaplan–Meier method, and differences between groups were assessed with the log-rank test. Parameters showing statistical significance for OS in univariate Cox models were further assessed using multivariate Cox models. Hazard ratios (HRs) and 95% CIs were subsequently calculated. All statistical tests used in this study were two-sided, and a *P* value < 0.05 was considered statistically significant.

## Results

### Patient characteristics and survival outcome

**Fig. 1:**
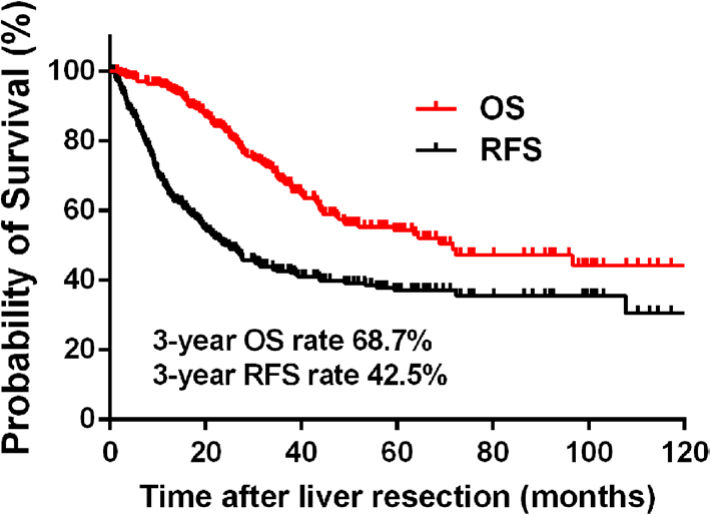
Overall survival (OS) and recurrence-free survival (RFS) rates for patients with colorectal liver oligometastases (CLOM) who underwent curative liver resection

Table 1 summarizes the patient demographics, characteristics of primary tumors and liver metastases, and treatment information for the study population. The median age of the 307 patients was 57.5 years (range 25–82 years), with 203 male patients (66.1%) and 104 female patients (39.9%). Among them, 42 patients (13.7%) were hepatitis B virus (HBV) surface antigen positive. The patients were followed for a median of 31.7 months (range 6.0–126.0 months). Overall, 106 (34.5%) patients died from the disease, 48 (15.6%) patients were alive with tumors, and 153 (49.8%) were alive without tumors at the end of follow-up. As shown in Fig. 1, the 1- and 3-year OS rates were 95.0 and 68.7%, respectively, while the 1- and 3-year RFS rates were 65.9 and 42.5%, respectively. The 3-year RFS and OS rates in the subgroups of patients were presented in Table 2.

**Table 1:** Patient demographics, tumor characteristics, and treatment in the total study population

Parameters	Total patients (*n*, %)
Patient characteristics	
Median age (year)	57.5 (25–82)
Age, years	
≤ 60	188 (61.2)
> 60	119 (38.8)
Sex	
Male	203 (66.1)
Female	104 (33.9)
HBV infection	
Negative	255 (86.3)
Positive	42 (13.7)
Primary tumor characteristics	
Primary tumor location	
Right-side colon	71 (23.1)
Left-side colon	119 (33.8)
Rectum	117 (38.1)
Primary tumor differentiation	
Well to moderate	235 (76.5)
Poor	72 (23.5)
T stage^a^	
1	3 (1.1)
2	24 (8.5)
3	158 (56.0)
4	97 (34.4)
N stage^b^	
0	117 (42.4)
1	99 (35.9)
2	60 (21.7)
Liver metastasis characteristics	
Timing of metastasis	
Synchronous	204 (66.4)
Metachronous	103 (33.6)
Number of metastatic tumors	
1	162 (52.8)
2	81 (26.4)
3	33 (10.7)
4	23 (7.5)
5	8 (2.6)
Metastasis diameter (cm)^c^	
Median (range)	2.5 (0.3–12)
≤ 3	204 (66.4)
> 3	99 (32.2)
Tumor distribution	
Unilobar	230 (74.9)
Bilobar	77 (25.1)
KRAS status^d^	
Wild type	49 (72.1)
Mutation type	19 (27.9)
Treatment characteristics	
Median resection margin (cm)^e^	0.5 (0–3.5)
Intraoperative RFA	
Yes	31 (10.1)
No	276 (89.9)
Preoperative chemotherapy	
Oxaliplatin-based regimen	91 (12.1)
Irinotecan-based regimen	37 (29.6)
5-Fluorouracil alone	8 (2.6)
No	171 (55.7)
Preoperative targeted therapy	
Bevacizumab	17 (5.5)
Cetuximab	13 (4.2)
No	207 (90.2)
Adjuvant chemotherapy	
Oxaliplatin-based regimen	48 (15.6)
Irinotecan-based regimen	160 (52.1)
5-Fluorouracil alone	18 (5.9)
No	81 (26.4)

*HBV* hepatitis B virus, *RFA* radiofrequency ablation

^a^Data of 282 patients were available

^b^Data of 276 patients were available

^c^Data of 303 patients were available

^d^Data of 68 patients were available

^e^Data of 181 patients were available

**Table 2:** 3-year recurrence-free survival and overall survival in the patients with colorectal liver oligometastases stratified by clinical characteristics

Parameters	3-year RFS rate (%)	3-year OS rate (%)
Age, years		
≤ 60	42.8	72.2
> 60	41.9	64.3
Sex		
Male	47.3	66.8
Female	40.0	72.0
Primary tumor location		
Colon	43.3	73.1
Rectum	41.1	62.3
Primary tumor differentiation		
Well to moderate	44.6	71.5
Poor	35.6	58.9
T stage		
1–3	46.0	72.5
4	38.4	65.7
N stage		
0	55.6	75.3
1–2	32.9	65.1
Timing of metastasis		
Synchronous	43.4	67.3
Metachronous	40.7	70.7
Number of metastatic tumors		
1	54.7	77.0
> 1	29.1	59.4
Metastases diameter (cm)		
≤ 3	47.5	70.9
> 3	33.8	67.0
Tumor distribution		
Unilobar	49.7	73.9
Bilobar	21.3	53.1
Preoperative CEA (ng/ml)		
≤ 10	45.7	65.4
> 10	38.6	70.9
Preoperative CA199 (U/ml)		
≤ 35	42.5	68.0
> 35	41.3	65.1
Resection margin (cm)		
≤ 0.5	29.7	66.0
> 0.5	49.0	73.8
Intraoperative RFA		
Yes	16.0	59.5
No	45.4	69.7
Perioperative chemotherapy		
Yes	50.6	69.4
No	40.8	64.4
Postoperative recurrence		
Yes	–	49.2
No	–	100

*RFS* recurrence-free survival, *OS* overall survival, *CEA* carcinoembryonic antigen, *CA199* cancer antigen (CA) 199, *RFA* radiofrequency ablation

### Association of recurrence and overall survival

**Fig. 2:**
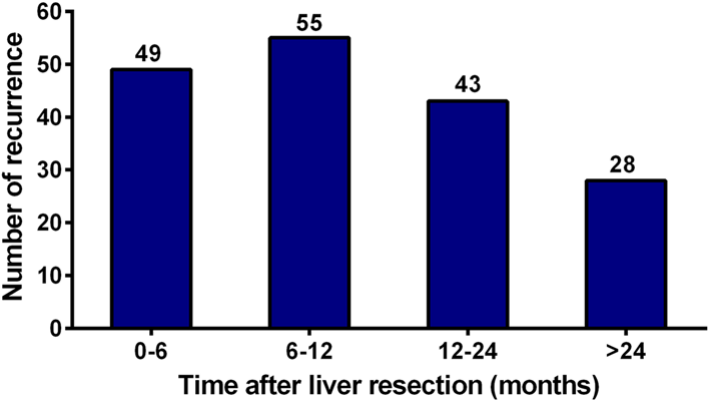
Quantification of the time of recurrence following curative liver resection for patients with colorectal liver oligometastases (CLOM)

**Fig. 3:**
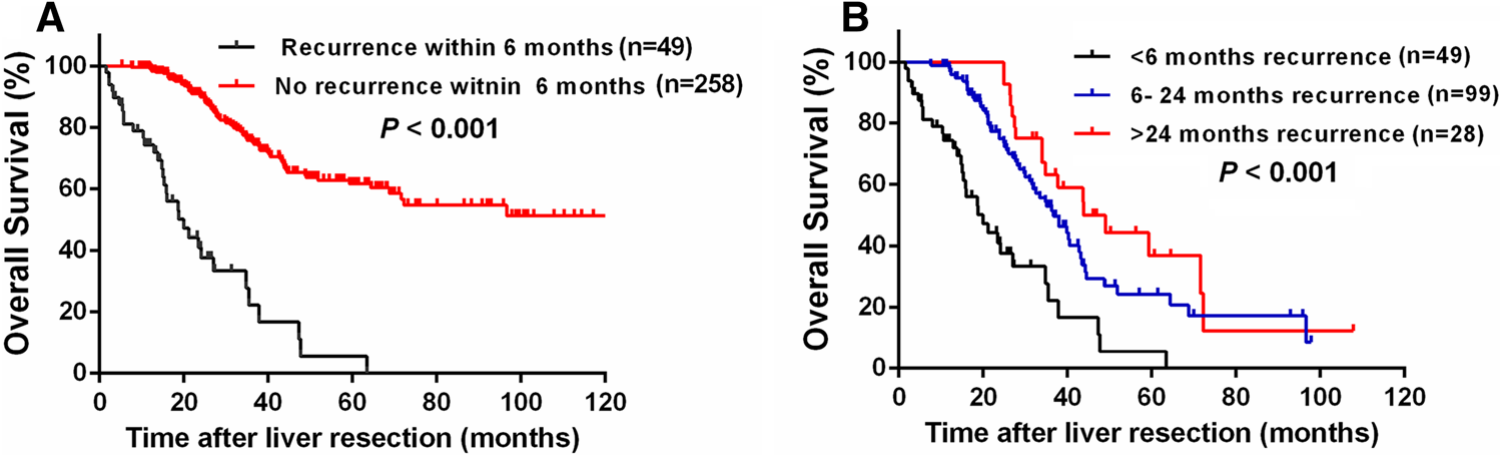
Comparison of overall survival (OS) after curative liver resection for patients with **a** early recurrence (< 6 months) and non-early recurrence (≥ 6 months or no recurrence) and **b** early recurrence (< 6 months), moderate recurrence (6–24 months), and late recurrence (> 24 months)

As shown in Fig. 2, recurrences were noted in 176 (57.3%) patients, including 49 (27.8%) early recurrences, 99 (56.3%) middle recurrences, and 28 (15.9%) late recurrences. Survival was reduced in patients with early recurrence compared to those without early recurrence (3-year OS rate 22.3 vs. 75.8%, *P* < 0.001, Fig. 3a). Likewise, patients with early recurrence showed the poorest 3-year OS rate compared to those with middle or late recurrence (22.3 vs. 52.8% vs. 63.2%, *P* < 0.001, Fig. 3b). Univariate analysis revealed that early recurrence (HR 7.121; 95% CI 4.608–11.004; *P* < 0.001), multiple metastatic tumors (HR 1.715; 95% CI 1.167–2.521; *P* = 0.006), metastases diameter > 3 cm (HR 1.607; 95% CI 1.085–2.378; *P* = 0.018), and bilobar liver metastases (HR 1.726; 95% CI 1.145–2.601; *P* = 0.009) were significantly associated with worse 3-year OS rates. In the multivariate Cox model, early recurrence (HR 6.282; 95% CI 3.980–9.915; *P* < 0.001) and multiple metastatic tumors (HR 1.542; 95% CI 1.039–2.288; *P* = 0.031) were identified as independent predictors of 3-year OS (Table 3).

**Table 3:** Univariate and multivariate analyses of risk factors for overall survival in patients with colorectal liver oligometastases after curative liver resection

Parameters	Univariate		Multivariate	
	HR (95% CI)	*P* value	HR (95% CI)	*P* value
Age (≤ 60 years vs. > 60 years)	1.439 (0.980–2.111)	0.063		
Gender (male vs. female)	1.192 (0.795–1.788)	0.396		
Primary tumor location (rectum vs. colon)	1.459 (0.996–2.137)	0.052		
Primary tumor differentiation (poor vs. well to moderate)	1.335 (0.862–2.068)	0.195		
T stage (4 vs. 1–3)	1.162 (0.765–1.766)	0.481		
N stage (positive vs. negative)	1.474 (0.967–2.248)	0.071		
Timing of metastasis (synchronous vs. metachronous)	1.578 (0.416–5.981)	0.503		
Number of metastatic tumors (> 1 vs. 1)	1.715 (1.167–2.521)	0.006	1.542 (1.039–2.288)	0.031
Metastases diameter (> 3 vs. ≤ 3 cm)	1.607 (1.085–2.378)	0.018		
Tumor distribution (bilobar vs. unilobar)	1.726 (1.145–2.601)	0.009		
Preoperative CEA (> 10 vs. ≤ 10 ng/ml)	1.045 (0.706–1.546)	0.826		
Preoperative CA199 (> 35 vs. ≤ 35 U/ml)	1.305 (0.864–1.969)	0.205		
Resection margin (> 0.5 vs. ≤ 0.5 cm)	0.703 (0.429–1.151)	0.161		
Intraoperative RFA (yes vs. no)	0.658 (0.368–1.178)	0.159		
Perioperative chemotherapy (yes vs. no)	1.019 (0.598–1.735)	0.945		
Early recurrence (yes vs. no)	7.121 (4.608–11.004)	< 0.001	6.282 (3.980–9.915)	< 0.001

*HR* hazard ratio, *CI* confidence interval, *CEA* carcinoembryonic antigen, CA199 cancer antigen (CA) 199, *RFA* radiofrequency ablation

### Risk factors predicting early recurrence

In the univariate analysis, patients with a node-positive primary tumor (72.7 vs. 27.3%; *P* = 0.027) and metastatic diameter > 3 cm (53.3 vs. 46.7%; *P* = 0.001) showed significantly higher chances of early recurrence (Table 3). In the multivariate logistic analysis, a node-positive primary tumor (OR 2.316; 95% CI 1.097–4.892; *P* = 0.028) and metastatic diameter > 3 cm (OR 2.560; 95% CI 1.290–5.078; *P* = 0.007) were identified as independent risk factors for early recurrence after CLOM resection (Table 4).

**Table 4:** Univariate and multivariate analyses of risk factors for early recurrence

Parameters	Early recurrence (*n*, %)	Non-early recurrence (*n*, %)	Univariate	Multivariate	
			*P* value	OR (95% CI)	*P* value
Age, years			0.200		
≤ 60	26 (53.1)	162 (62.8)			
> 60	23 (46.9)	96 (37.2)			
Sex			0.895		
Male	32 (65.3)	171 (66.3)			
Female	17 (34.7)	87 (33.7)			
Hepatitis B virus infection			0.750		
Negative	43 (87.8)	222 (86.0)			
Positive	6 (12.2)	36 (14.0)			
Primary tumor location			0.790		
Right-side colon	13 (26.5)	58 (22.5)			
Left-side colon	19 (38.8)	100 (38.8)			
Rectum	17 (34.7)	100 (38.8)			
Primary tumor differentiation			0.356		
Well to moderate	35 (71.4)	200 (77.5)			
Poor	14 (28.6)	58 (22.5)			
T stage^a^			0.441		
1–3	26 (60.5)	159 (66.5)			
4	17 (39.5)	80 (33.5)			
N stage^b^			0.027	2.316 (1.097–4.892)	0.028
0	12 (27.3)	105 (45.3)			
1–2	32 (72.7)	127 (54.7)			
Timing of metastasis			0.607		
Synchronous	31 (63.3)	173 (67.1)			
Metachronous	18 (36.7)	85 (32.9)			
Number of metastatic tumors			0.271		
1	21 (42.9)	141 (54.7)			
2–3	21 (42.9)	93 (36.0)			
4–5	7 (14.3)	24 (9.3)			
Metastases diameter (cm)^c^			0.001	2.560 (1.290–5.078)	0.007
≤ 3	21 (46.7)	183 (70.9)			
> 3	24 (53.3)	75 (29.1)			
Tumor distribution			0.090		
Unilobar	32 (65.3)	198 (76.7)			
Bilobar	17 (34.7)	60 (23.3)			
Preoperative CEA (ng/ml)^d^			0.249		
≤ 10	23 (47.9)	139 (57.0)			
> 10	25 (52.1)	105 (43.0)			
Preoperative CA199 (U/ml)^e^			0.284		
≤ 35	30 (62.5)	166 (70.3)			
> 35	18 (37.5)	70 (29.7)			
KRAS status^f^			0.852		
Wild type	14 (73.7)	35 (71.4)			
Mutation	5 (26.3)	14 (28.6)			
Resection margin (cm)^g^			0.301		
0–0.5	18 (60.0)	75 (49.7)			
> 0.5	12 (40.0)	76 (50.3)			
Intraoperative RFA			0.302		
Yes	7 (14.3)	24 (9.3)			
No	42 (85.7)	234 (90.7)			
Preoperative chemotherapy			0.097		
Yes	27 (55.1)	109 (42.2)			
No	22 (44.9)	149 (57.8)			
Adjuvant chemotherapy			0.705		
Yes	35 (71.4)	191 (74.0)			
No	14 (28.6)	67 (26.0)			
Perioperative chemotherapy			0.284		
Yes	43 (87.8)	210 (81.4)			
No	6 (12.2)	48 (18.6)			

*OR* odds ratio, *CI* confidence interval, *HBV* hepatitis B virus, *CEA* carcinoembryonic antigen, *CA199* cancer antigen (CA) 199, *RFA* radiofrequency ablation

^a^Data of 282 patients were available

^b^Data of 276 patients were available

^c^Data of 303 patients were available

^d^Data of 292 patients were available

^e^Data of 284 patients were available

^f^Data of 68 patients were available

^g^Data of 181 patients were available

### Recurrence sites and treatment between early recurrence and later recurrence

As shown in Table 5, among 49 early recurrence patients, 28 (57.1%) developed intrahepatic recurrence, while 15 (30.6%) developed extrahepatic recurrence, with 6 (12.2%) patients lacking information on the recurrence sites. In 127 later recurrence patients, 51 (40.2%) patients developed intrahepatic recurrence, while 25 (19.6%) patients were lacking information on the recurrence sites. The rate of a single recurrence site was similar between the patients developing early recurrence and later recurrence (83.7 vs. 74.5%, *P* = 0.227). There were no significant differences in the rate of acceptance of recurrence treatment, palliative chemotherapy, and radiofrequency ablation (RFA) between the two groups. Nevertheless, the salvage liver resection rate was significantly lower in patients with early recurrence than in those with later recurrence (4.1 vs. 19.7%, *P* = 0.010).

**Table 5:** Comparison of recurrence sites and treatment between patients with early and later recurrence

Parameters	Early recurrence (*n* = 49, %)	Later recurrence (*n* = 127, %)	*P* value
Recurrence site			
Single sites	36 (83.7)	76 (74.5)	0.227
Liver	28	51	
Lung	3	16	
Peritoneum	4	8	
Others	1	1	
Multiple sites	7 (16.3)	26 (25.5)	
Unknown	6	25	
Recurrence treatment pattern			
Acceptance of recurrence treatment	35 (71.4)	84 (66.1)	0.502
Salvage liver resection	2 (4.1)	25 (19.7)	0.010
Palliative chemotherapy	22 (44.9)	57 (44.9)	0.998
Radiofrequency ablation	16 (32.7)	28 (22.0)	0.145

## Discussion

Accumulating evidence has shown that fewer liver metastases are significantly associated with less recurrence, thus translating to better survival after R0 hepatic resection for CRLM patients (Chan et al. 2014; Fong et al. 1999; Sasaki et al. 2016; Zhang et al. 2010). Characterized by liver-only metastases with a limited number of lesions (≤ 5 metastases), liver oligometastases have been correspondingly identified to indicate low-risk CLRM patients, who may achieve a favorable survival outcome after curative liver metastasis treatment (Lu et al. 2016; Takeda et al. 2016; Weiser et al. 2013). The previous studies have reported that the 3-year OS rate in patients with unresectable and widespread systemic CRLM was lower than 50% (Chafai et al. 2005; Folprecht et al. 2014; Li et al. 2014), while the current study showed that the 3-year OS rate was 68.7%, with a 3-year RFS rate of 42.5%, in CLOM patients undergoing complete resection of total lesions.

Despite these excellent outcomes, recurrence remains an intractable problem for treating CLOM patients. Our previous study found that even when the primary and metastatic tumors were resected, up to 50% of patients with CLOM experienced disease recurrence postoperatively. However, the high-risk event, early recurrence, was not definitively identified and investigated in that study (Lu et al. 2016). Herein, our current study showed that 16.0% (49/307) of patients with CLOM developed early recurrence after liver resection. Among them, approximately 50% of the recurrences occurred within 3 months postoperatively. In addition, intrahepatic recurrence was the most common recurrence pattern in early recurrence.

Early recurrence was identified as an independent risk factor for poor long-term survival and was recognized as the leading cause of death within 5 years of curative resection of CRLM (Kaibori et al. 2012; Vigano et al. 2014; Yamashita et al. 2011). In the current investigation, we focused on early recurrence and its influence on prognosis in CLOM patients. Although CLOM represents a relatively non-aggressive tumor biology with limited widespread metastatic capacity (Reyes and Pienta 2015), our study revealed that patients with early recurrence faced a poor outcome. Hence, the identification of risk factors and methods to screen out high-risk subgroups for early recurrence are urgently needed to guide individual treatment.

In CRLM patients undergoing liver resection, early recurrence has been confirmed to be associated with more aggressive diseases, such as synchronous, multiple and large-mass metastases, advanced T and N staging of primary tumors, inadequate surgical resection, and failure of systemic therapy (Bhogal et al. 2015; Imai et al. 2016; Jung et al. 2016; Malik et al. 2007; Vigano et al. 2014. For the first time, our study demonstrated that in CLOM patients undergoing curative liver resection, the independent risk factors for early recurrence were node-positive primary tumors and a metastasis diameter > 3 cm. Unlike the results of the studies by Malik et al. (2007) and Yamashita et al. (2011), the number of liver metastases was not identified as a risk factor in these selected patients. We considered that the actual predictive effect of the number of liver metastases for early recurrence might not be easily determined in CLOM patients with complete tumor resection because of the limited burden of metastatic tumors. Based on these results, detailed preoperative comprehensive measurement of the disease is urgently needed, as this may help oncologists select patients with different risks of early recurrence. Once patients are diagnosed with CLOM with advanced N staging of the primary tumor and large-mass liver metastases, the limited benefit of surgery and high risk of early recurrence in this cohort of patients should be carefully taken into consideration. For these patients, intensive chemotherapy has been proposed to increase the control of micrometastatic disease and, more importantly, to provide a test for chemo responsiveness, which could further identify aggressive disease and select good candidates for subsequent surgery (Allen et al. 2003; Power and Kemeny 2010). On the other hand, a non-surgical strategy might be an effective alternative to immediate surgery as the first-line treatment. For instance, RFA has been proposed as an effective ablative technology to provide survival benefits comparable to surgical resection for patients with resectable CRLM (Ko et al. 2014; Otto et al. 2010). Taken together, we suggest the combination of intensive chemotherapy and local ablation as the first-line treatment for these high-risk patients.

It has been noted that subsequent treatment might be a crucial factor to prolong the survival of patients with early recurrence (Lan et al. 2014; Vigano et al. 2014). Although salvage resection could prolong long-term survival for patients with liver recurrence, the secondary resection rate was significantly lower in patients with early recurrence than in those with late recurrence (Imai et al. 2016). Similarly, in our study, less than 5% of patients with early recurrence were able to receive salvage liver resection, and the resection rate was significantly lower for patients with early recurrence than for those with later recurrence (4.1 vs. 19.7%, *P* = 0.010). Within a shorter postoperative period, several factors could impede the performance of surgical resection, including worsened condition status, potential surgical complications, and development of unresectable metastases. As a result, the low probability of salvage liver resection for early recurrence disease could contribute to the poorer long-term survival for these patients. Nevertheless, early recurrence should not be considered a hopeless situation. Early engagement and communication among members of an MDT that includes surgeons, medical oncologists, radiation oncologists, and other specialists are needed to combine local ablative and systemic treatment to optimize the chance for a cure (Weiser et al. 2013).

Some limitations of this study should be acknowledged. First, this retrospective study included an uncontrolled methodology and a limited number of patients from a single institution. Therefore, the findings need to be validated in a larger prospective cohort of patients. Second, the short duration of follow-up time was insufficient to evaluate 5-year survival outcomes and exactly determine which patients experienced late disease recurrence. In addition, the impact of chemotherapy on early recurrence was difficult to evaluate in this retrospective study. Moreover, preoperative treatment selectively given to patients with more advanced disease might interfere with its real therapeutic effect. At the same time, the regimen and administration duration of perioperative chemotherapy might be related to the occurrence of early recurrence, which was not analyzed in the current study. Moreover, the data for molecular biomarkers such as KRAS, NRAS, BRAF, and PIK3CA mutations and microsatellite instability (MSI) status were not available for the majority of patients in this study. Thus, future studies should include an evaluation of these molecular biomarkers.

## Conclusion

Early recurrence occurred in 16.0% of CLOM patients, even those undergoing curative liver resection, and was identified as the independent predictor of poor prognosis. The risk factors for predicting early recurrence included the presence of a node-positive primary tumor and metastases diameter > 3 cm. Our study results suggest that early recurrence should be investigated in the routine treatment of CLOM patients before liver resection. Detailed preoperative comprehensive measurements might be needed to stratify high-risk patients, and non-surgical treatment for early recurrence might represent an effective alternative.
